# GPER Mediates Cardiotropic Effects in Spontaneously Hypertensive Rat Hearts

**DOI:** 10.1371/journal.pone.0069322

**Published:** 2013-08-09

**Authors:** Ernestina Marianna De Francesco, Tommaso Angelone, Teresa Pasqua, Marco Pupo, Maria Carmela Cerra, Marcello Maggiolini

**Affiliations:** 1 Department of Pharmacy, Health and Nutritional Sciences, University of Calabria, Rende, Cosenza, Italy; 2 Department of Biology, Ecology and Earth Sciences, University of Calabria, Rende, Cosenza, Italy; University of Padova, Italy

## Abstract

Estrogens promote beneficial effects in the cardiovascular system mainly through the estrogen receptor (ER)α and ERβ, which act as ligand-gated transcription factors. Recently, the G protein-coupled estrogen receptor (GPER) has been implicated in the estrogenic signaling in diverse tissues, including the cardiovascular system. In this study, we demonstrate that left ventricles of male Spontaneously Hypertensive Rats (SHR) express higher levels of GPER compared to normotensive Wistar Kyoto (WKY) rats. In addition, we show that the selective GPER agonist G-1 induces negative inotropic and lusitropic effects to a higher extent in isolated and Langendorff perfused hearts of male SHR compared to WKY rats. These cardiotropic effects elicited by G-1 involved the GPER/eNOS transduction signaling, as determined by using the GPER antagonist G15 and the eNOS inhibitor L-NIO. Similarly, the G-1 induced activation of ERK1/2, AKT, GSK3β, c-Jun and eNOS was abrogated by G15, while L-NIO prevented only the eNOS phosphorylation. In hypoxic Langendorff perfused WKY rat heart preparations, we also found an increased expression of GPER along with that of the hypoxic mediator HIF-1α and the fibrotic marker CTGF. Interestingly, G15 and L-NIO prevented the ability of G-1 to down-regulate the expression of both HIF-1α and CTGF, which were found expressed to a higher extent in SHR compared to WKY rat hearts. Collectively, the present study provides novel data into the potential role played by GPER in hypertensive disease on the basis of its involvement in myocardial inotropism and lusitropism as well as the expression of the apoptotic HIF-1α and fibrotic CTGF factors. Hence, GPER may be considered as a useful target in the treatment of some cardiac dysfunctions associated with stressful conditions like the essential hypertension.

## Introduction

Compelling experimental evidence and epidemiological studies support the idea that the different impact of cardiovascular diseases between women and men may be related, at least in part, to the beneficial effects exerted by estrogens [1]–[4]. Classically, the estrogen receptor (ER)α and ERβ mediate the biological responses to estrogens acting as ligand-gated transcription factors [5], [6], however membrane associated receptors are also involved by estrogens in triggering gene transcription and physiological functions [7]–[9]. In this regard, it has been shown that the novel estrogen receptor named GPR30/GPER mediates estrogenic signals [10]–19 activating diverse transduction cascades like the extracellular signal-related kinase (ERK), the phosphatidylinositol-3-kinase (PI3K)/AKT pathway, Ca2+ mobilization and cAMP production [20]–[23]. Among numerous tissues, GPER was detected in the cardiovascular system suggesting that it may play a physiological role in the regulation of vascular and myocardial function [24]. In line with these findings, we have demonstrated in the isolated and perfused rat hearts that the selective GPER ligand G-1 induces negative inotropic responses through the ERK and the endothelial Nitric Oxide Synthase (eNOS) transduction pathways [25]. In addition, G-1 improved the functional recovery and reduced the infarct size following ischemia and reperfusion (I/R) in Sprague Dawley rat hearts [26]. G-1 lowered also the mean arterial pressure in normotensive rats [27] and the systolic blood pressure in ovariectomized female mRen2 Lewis rats [28]. In cardiomyocytes exposed to hypoxia, GPER and the fibrotic mediator Connective Tissue Growth Factor (CTGF) were found up-regulated together with the key factor mediating the adaptive response to low oxygen tension, such as the Hypoxia Inducible factor-1 (HIF-1) [29]. Notwithstanding the potential ability of GPER in mediating the beneficial effects of estrogens in the cardiovascular system, its role in the essential hypertension and cardiac remodeling remains to be fully elucidated. In the present study, using as a model system the Spontaneously Hypertensive Rats (SHR), we provide novel insights into the mechanisms through which GPER may elicit a cardioprotective action in stressful conditions like essential hypertension.

## Materials and Methods

### Animals

Male Wistar Kyoto (WKY) and Spontaneous Hypertensive Rats (SHR) (450–500 g; 20 weeks old; Harlan Laboratories s.r.l. Udine, Italy) ad libitum fed with a standard diet and with free water access. Blood pressure (BP), measured before each experiment by tail-cuff method, was: WKY: Systolic BP = 127.5±5.4 mmHg and Diastolic BP = 83.5±4.5 mmHg; SHR: Systolic BP = 181.9±7.9 mmHg and Diastolic BP = 124.3±6.2 mmHg. Heart weights were: WKY: 1,75±0,18 g; SHR: 2,25±0,2 g.

### Ethics Statement

All procedures conformed to the Guiding Principles in the Care and Use of Animals (US National Institutes of Health, Publication No. 85-23, revised 1996) and the project were supervised and approved by the ethics committee of the Department of Pharmacy, Health and Nutritional Sciences, University of Calabria. All surgery was performed under anesthesia and all efforts were made to minimize suffering.

### Langendorff perfused rat heart

Rats were anaesthetized by i.p. injection of ethyl carbamate (2 g/kg body weight). Hearts were then dissected out and mounted on a Langendorff apparatus for perfusion with a Krebs-Henseleit solution (KHs) composed of (in mM) NaCl 113, KCl 4.7, NaHCO_3_ 25, MgSO_4_ 1.2, CaCl_2_ 1.8, KH_2_PO_4_ 1.2, glucose 11, mannitol 1.1, Na-pyruvate 5 and gassed with 95%O_2_-5%CO_2_ (pH 7.4, 37°C), or gassed with 50%O_2_-45%N_2_-5%CO_2_ (pH 7.4, 37°C) for hypoxic stimulation. KHs was delivered at a constant flow-rate of 12 mL/min [30]. All hearts were perfused for a 15 min equilibration period. After the equilibration period, the hearts (n = 5) were randomly assigned to one of the following groups: Group 1 (control): KH; Group 2: KH plus G-1; Group 3: KH in the presence of G-1 plus G15; Group 4: KH in the presence of G-1 plus L-NIO; Group 5: KH gassed with 50%O_2_-45%N_2_-5%CO_2_. Cardiac and hemodynamic parameters were evaluated as previously described [31], [32]. Briefly, to measure left ventricular pressure, a water-filled latex balloon connected to a BLPR gauge (WRI, Inc. USA) was inserted through the mitral valve into the left ventricle to allow isovolumic contractions and to continuously record mechanical parameters. The balloon was progressively filled with water to obtain an initial left ventricular end diastolic pressure of 5 to 8 mmHg [30]. Hemodynamic parameters were assessed using a PowerLab data acquisition system and analyzed using Chart software (ADInstruments, Basile, Italy). Heart performance was evaluated from the Left Ventricular pressure (LVP, in mmHg) which is an index of contractile activity, the rate-pressure product (RPP: HR×LVP, in 104 mmHg×beats/min) which is an index of cardiac work, the maximal value of the first derivative of LVP (mmHg/sec) which is an index of the maximal rate of LV contraction, the time to Peak Tension of isometric twitch (Ttp) which is an assessement of inotropism. Lusitropism was determined by calculating the maximal rate of LVP decline -(LVdP/dT)max (mmHg/sec), the half time relaxation (HTR) (sec), which is the time required for tension to fall from the peak to 50% and T/-t ratio obtained by +(LVdP/dT)max/-(LVdP/dT)max. Mean CP was calculated by averaging values obtained during several cardiac cycle [30].

### Basal conditions

Cardiac performance was evaluated for inotropism by analyzing the left ventricular pressure (LVP, in mmHg) (index of contractility), the rate-pressure product (RPP) (index of cardiac work), the maximal value of the first derivative of LVP (+(LVdP/dt)max) (mmHg/sec) (index of the maximal rate of left ventricular contraction), Ttp (Time to Peak Tension of isometric twitch), and for lusitropism by analyzing the maximal rate of left ventricular pressure decline of LVP (−(LVdP/dt)max) (mmHg/sec), the half time relaxation (HTR) (sec) (time required for tension to fall from the peak to 50%) and T/-t ratio obtained by +(LVdP/dt)max/−(LVdP/dt)max. Mean CP (mmHg) was the average of values obtained during several cardiac cycles [30].

### Experimental protocols

#### G-1 stimulated preparations

Preliminary experiments (data not shown) obtained by repetitive exposure of each heart to one concentration of G-1 (1 nmol/L) revealed no desensitization. Thus, concentration-response curves were generated by perfusing the hearts (WKY and SHR) with KHs plus increasing concentrations of G-1 (from 1 pmol/L to 10 nmol/L) for 10 min. To confirm that G-1 specifically activates GPER, hearts were perfused with the selective GPER antagonist G15 (100 nmol/L) alone for 10 min and then exposed to G15 (100 nmol/L) in combination with increasing concentrations of G-1 (from 1 pmol/L to 10 nmol/L).

#### eNOS involvement

To verify the involvement of eNOS in the GPER-mediated cardiotropic effects, hearts were perfused with L-NIO (10 µmol/L) alone for 10 min and then in presence of L-NIO (10 µmol/L) in combination with increasing concentrations of G-1 (from 1 pmol/L to 10 nmol/L).

#### Hypoxia stimulated preparations

Hearts were dissected out and connected to a Langendorff apparatus for perfusion with a Krebs-Henseleit solution gassed with 50%O_2_-45%N_2_-5%CO_2_. Left ventricular pressure, heart rate and coronary flow were monitored throughout the perfusion protocol.

At the end of the perfusions, ventricles were excised and immediately processed for RNA and protein extraction.

### Reagents

1-(4-(-6-Bromobenzol(1,3)diodo-5-yl)3a,4,5,9b-tetrahidro-3H-cyclopenta(c-)quinolin-8yl)ethanone (G-1) was purchased from Merck KGaA (Frankfurt, Germany). G15 was a kind gift from Dr Prossnitz (University of New Mexico). L-N5-(1-iminoethyl)ornithine (L-NIO) was purchased from Sigma-Aldrich (Milan, Italy). Reagents were dissolved in dimethylsulfoxide. Preliminary experiments showed that the presence of equivalent amounts of DMSO in KHs solution did not modify basal cardiac performance.

### Ventricular tissue homogenates preparation and gene expression studies

Gene expression was evaluated by real-time PCR. Briefly, the left ventricles were excised, homogenized with a motor-driven homogenizer and total RNA was isolated using the Trizol reagent (Invitrogen, Milan, Italy), according to the manufacturer's instructions. RNA was quantified spectrophotometrically and quality was checked by electrophoresis through agarose gels stained with ethidium bromide. Only samples that were not degraded and showed clear 18 and 28 S bands under UV light were used for RT-PCR. Total cDNA was synthesized from the RNA by reverse transcription using the murine leukemia virus reverse transcriptase (Invitrogen, Milan, Italy) following the protocol provided by the manufacturer. The expression of selected genes was quantified by real-time PCR using the Step OneTM sequence detection system (Applied Biosystems Inc., Milan, Italy), following the manufacturer's instructions. Gene-specific primers were designed using Primer Express version 2.0 software (Applied Biosystems Inc., Milan, Italy) and are as follows: ERα Fwd: 5′-AGGAGACTCGCTACTGTGCTG-3′ and Rev: 5′-ATCATGCCCACTTCGTAACAC-3′; ERβ Fwd: 5′-CACTGCACTTCCCAGGAGTCA-3′ and Rev: 5′-AACTTGGCATTCGGTGGTACAT-3′; GPER Fwd: 5′-TCTACCTACCCTCCCGTGTGG-3′ and Rev:5′-AGGCAGGAGAGGAAGAGAGC-3′; HIF-1α Fwd: 5′-AACAAACAGAATCTGTCCTCAAAC C-3′ and Rev: 5′-CAGGTAATGGAGACATTG CCAG-3′; CTGF Fwd: 5′-AAGACCTGTGGGATGGGC-3′ and Rev: 5′-TGGTGCAGCCAGAAAGCTC-3′; 18S Fwd: 5′- TTTGTTGGTTTTCGGAACTGA -3′ and Rev: 5′- CGTTTATGGTCGGAACTACGA -3′. 18S expression was used as PCR amplification control. The relative gene expression levels were normalized to a calibrator that was chosen to be the WKY rat samples. Final results were expressed as n-fold differences in gene expression relative to 18S rRNA and calibrator, calculated following the ΔΔCt method as follows: n-fold = 2 ^−(ΔCt sample−ΔCt calibrator)^. The ΔCt values of the sample and calibrator were determined by subtracting the average cycle threshold (Ct) value of the 18S rRNA reference gene from the average Ct value of the different genes analyzed.

### Western Blot Analysis

To prepare lysates, ventricles from rat hearts (n = 5) were homogenized with a motor-driven homogenizer prior to extraction which was performed using 50 mM Hepes solution, pH 7.4, containing 1% (v/v) Triton X-100, 4 mM EDTA, 1 mM sodium fluoride, 0.1 mM sodium orthovanadate, 2 mM PMSF, 10 µg/ml leupeptin and 10 µg/ml aprotinin. Protein concentrations in the supernatant were determined according to the Bradford method. Tissue lysates (10–50 µg of protein) were electrophoresed through a reducing SDS/10% (w/v) polyacrylamide gel and electroblotted onto a nitrocellulose membrane. After the transfer, the membranes were stained with Red Poinceau to confirm equal loading and transfer. Membranes were blocked and incubated with primary polyclonal IgG antibody for HIF-1α (R&D Systems, Inc. Celbio, Milan, Italy), ERβ (Serotec, Milan, Italy), ERα (F-10), GPER (N-15), CTGF (L-20), phosphorylated ERK1/2 (E-4), ERK2 (C-14), phosphorylated-c-Jun Ser 73, c-Jun (N), p-AKT1/2/3 Ser 473-R, p- GSK-3β Ser 9, p-NOS3 Ser 1177, β-tubulin (H-235-2), AKT/1/2/3 (H-136), eNOS/NOS3 (B-5), GSK3β (4E95) and appropriate secondary HRP-conjugated antibodies, all purchased from Santa Cruz Biotechnology (DBA, Milan, Italy). The levels of proteins and phosphoproteins were detected with horseradish peroxidase-linked secondary antibodies and revealed using the Enhanced Chemiluminescence system (GE Healthcare, Milan, Italy).

### Statistics

Data are expressed as the mean±SD. Since each heart represents its own control, the statistical significance of differences within groups was assessed using one-way ANOVA. Comparison between groups was made by using a one-way analysis of variance (ANOVA) followed by the Bonferroni correction for post hoc t-tests. Differences were considered to be statistically significant for (○),(•),(□),(▪),(*),(§),(+) p<0.05.

## Results

### ERs and GPER expression in WKY and SHR heart tissues

**Figure 1:**
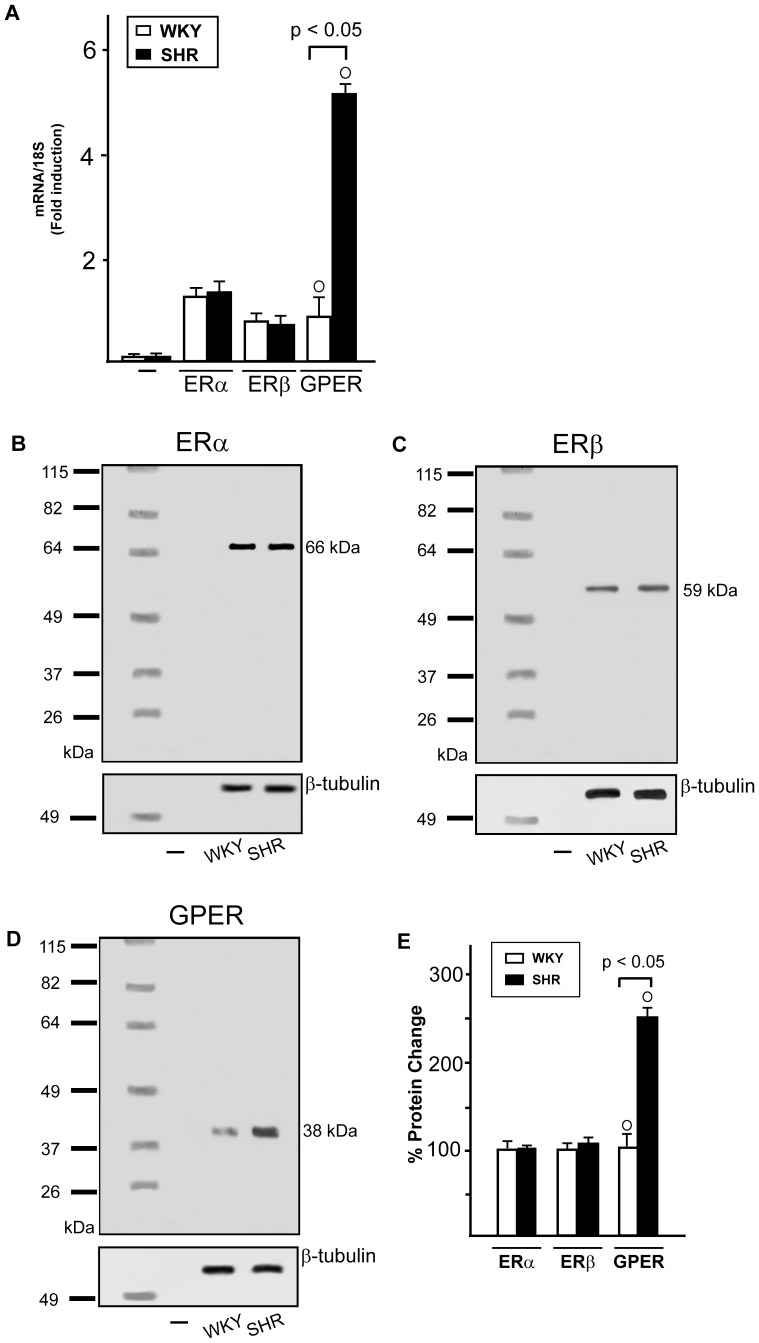
ERs expression in WKY and SHR left ventricular homogenates. (A) ERα, ERβ and GPER mRNA expression in WKY and SHR left ventricular tissue, as evaluated by Real Time PCR and normalization to 18S expression, PCR amplification in absence of cDNA was used as a control (-). Bars represent the mean±SD of 5 experiments for each group. (○) p<0.05 for the expression in SHR vs WKY. (B) ERα, (C) ERβ and (D) GPER protein expression in male WKY and SHR ventricular tissue, lysis buffer without proteins was used as a control (-), protein expressions were normalized to β-tubulin. (E) Densitometric analysis of the blots. Percentage changes were evaluated as mean±SD of 5 experiments for each group. (○) p<0.05 for the expression in SHR vs WKY.

In order to provide novel insight into the molecular mechanisms involved in the cardiac effects elicited by estrogens, we began our study evaluating the expression of ERα, ERβ and GPER in the left ventricles of male WKY and SHR homogenates. The mRNA expression levels of both ERα and ERβ were similar in WKY and SHR preparations, while GPER expression was found increased in the left ventricles of SHR with compared to WKY rats, as evaluated by Real time RT-PCR (Fig. 1A) and semi-quantitative PCR (data not shown) [33]. In line with these results, the protein expression of GPER was higher in SHR homogenates compared to WKY preparations (Fig. 1D), while the protein levels of ERα and ERβ were similar in WKY and SHR hearts (Fig. 1B,C).

### Cardiotropic effects of G-1 in WKY and SHR

#### Basal conditions

Cardiac parameters of WKY and SHR rats, obtained after 20 min equilibration, are in Table 1 and 2. Endurance and stability of the preparations, analyzed by measuring the performance variables every 10 min, showed that each heart was stable up to 180 min (data not shown).

**Table 1:** Basal cardiac parameters in WKY rat hearts.

LVP (mmHg)	HR (beats min^−1^)	EDVP (mmHg)	RPP (mmHg beats min^−1^)	+(LVdP/dt)_max_(mmHg s^−1^)	−(LVdP/dt)_max_(mmHg s^−1^)	Time to peak (s)	HTR (s)	T/-t (mmHg s^−1^)	CP (mmHg)	Pressure perfusion (mmHg)
89±3	280±7	5–8	2.5±0.1 10^4^	2492±129	−1663±70	0.08±0.01	0.05±0.01	1.498±1.84	63±3	100

For abbreviation see Material and Methods.

**Table 2:** Basal cardiac parameters in SHR hearts.

LVP (mmHg)	HR (beats min^−1^)	EDVP (mmHg)	RPP (mmHg beats min^−1^)	+(LVdP/dt)_max_(mmHg s^−1^)	−(LVdP/dt)_max_ (mmHg s^−1^)	Time to peak (s)	HTR (s)	T/-t (mmHg s^−1^)	CP (mmHg)	Pressure perfusion (mmHg)
106±4	330±12	5–8	3.5±0.510^4^	3290±109	−2595±54	0.12±0.01	0.07±0.01	1.2±1.65	110±8	100

For abbreviation see Material and Methods.

#### G-1 inotropic, lusitropic and coronary actions

**Figure 2:**
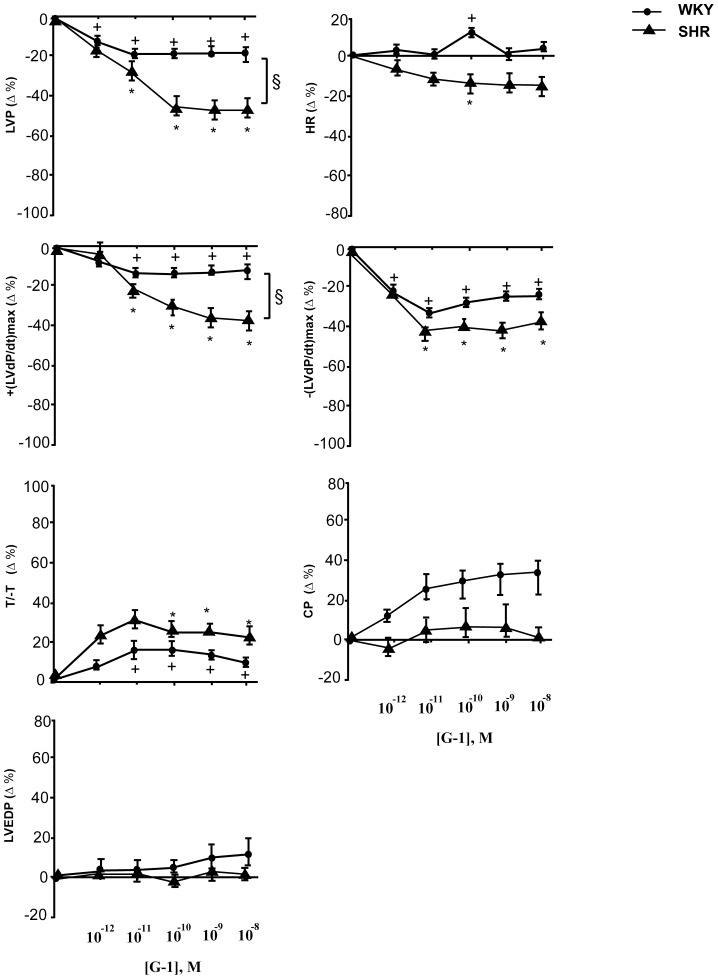
Negative inotropic and lusitropic effects induced by G-1. Dose-dependent response curves to G-1 (1 pmol/L÷10 nmol/L) on inotropic parameters: LVP and +(LVdP/dT)max, on lusitropic parameters: −(LVdP/dT)max, T/-t, CP and on LVEDP on Langendorff perfused male rat WKY and SHR heart preparations. For abbreviations and basal values see results. Percentage changes were evaluated as mean ± SD of 5 experiments. Significance of difference from control values (One way ANOVA); (*),(+) p<0.05; comparison between groups (ANOVA, Bonferroni's Multiple Comparison Test): (§) p<0.05. For abbreviations and basal values see results.

G-1 effects on basal cardiac performance were evaluated by exposing heart preparations to increasing concentrations of G-1 to generate concentration-response curves. Since G-1 effects remained stable until 15–20 min, cardiac parameters were measured at 10 min. On WKY hearts G-1 caused a concentration-dependent negative inotropic effect, showed by a decrement of LVP, significant starting from 1 pmol/L. G-1 also significantly reduced +(LVdP/dt)max from 10 pmol/L, without changing HR (Fig. 2). Analysis of the lusitropic changes revealed a reduction of -(LVdP/dt)max and an increase of T/-t from 10 pmol/L. G-1 also induced an increase in coronary pressure (Fig. 2). On SHR hearts increasing concentrations of G-1 induce a relevant negative inotropic effect which was significant from 10 pmol/L. It reduced LVP (49%) and +(LVdP/dt)max (46%), reaching a maximum at 10 nmol/L. G-1 also affects the lusitropic parameters, causing a concentration-dependent increment in T/-t and a decrement in -(LVdP/dt)max, without changing HR. G-1 treatment did not affect coronary pressure (Fig. 2) and LVEDP, an index of cardiac contracture.

### G-1 activated GPER signaling

**Figure 3:**
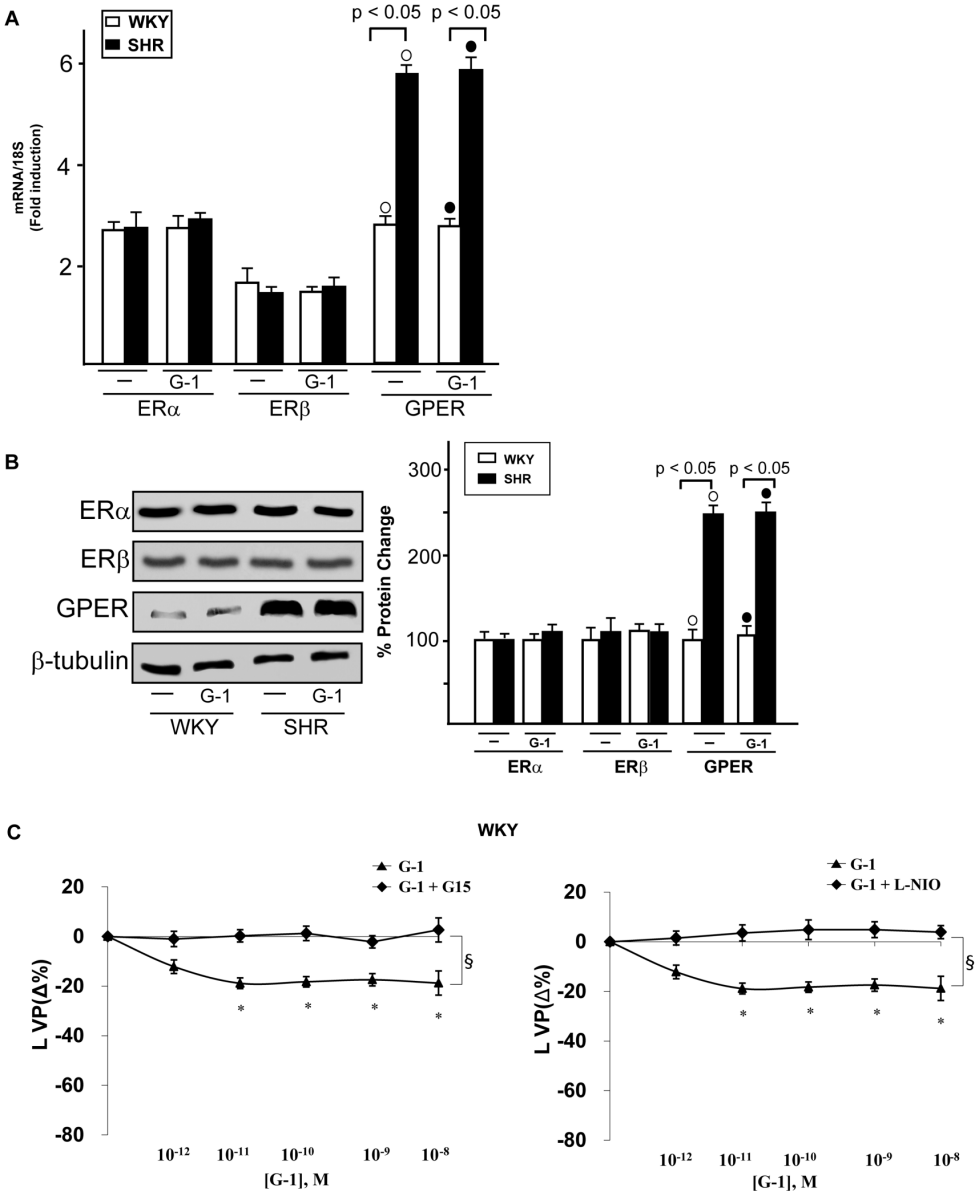
Involvement of eNOS in the cardiotropic actions induced by G-1. (A) ERα, ERβ and GPER mRNA expression in WKY and SHR left ventricular tissue perfused with vehicle (-) and 1 nmol/L G-1, as evaluated by Real Time PCR and normalization to 18S expression. Bars represent the mean±SD of 5 experiments for each group. (○), (•) p<0.05. (B) ERα, ERβ and GPER protein expression in male WKY and SHR left ventricular tissue perfused with vehicle (-) and 1 nmol/L G-1. Protein expressions were normalized to β-tubulin, percentage changes were evaluated as mean±SD of 5 experiments for each group. (○), (•) p<0.05. (C) LVP responses of isolated and perfused Langendorff rat heart preparations to G-1 alone (1 pmol/L÷10 nmol/L) and in combination with G15 (100 nmol/L) or L-NIO (10 µMol/L). Percentage changes were evaluated as mean±SD of 5 experiments for each group. Significance of difference from control values (One way ANOVA); (*) p<0.05; comparison between groups (ANOVA, Bonferroni's Multiple Comparison Test): (§) p<0.05.

**Figure 4:**
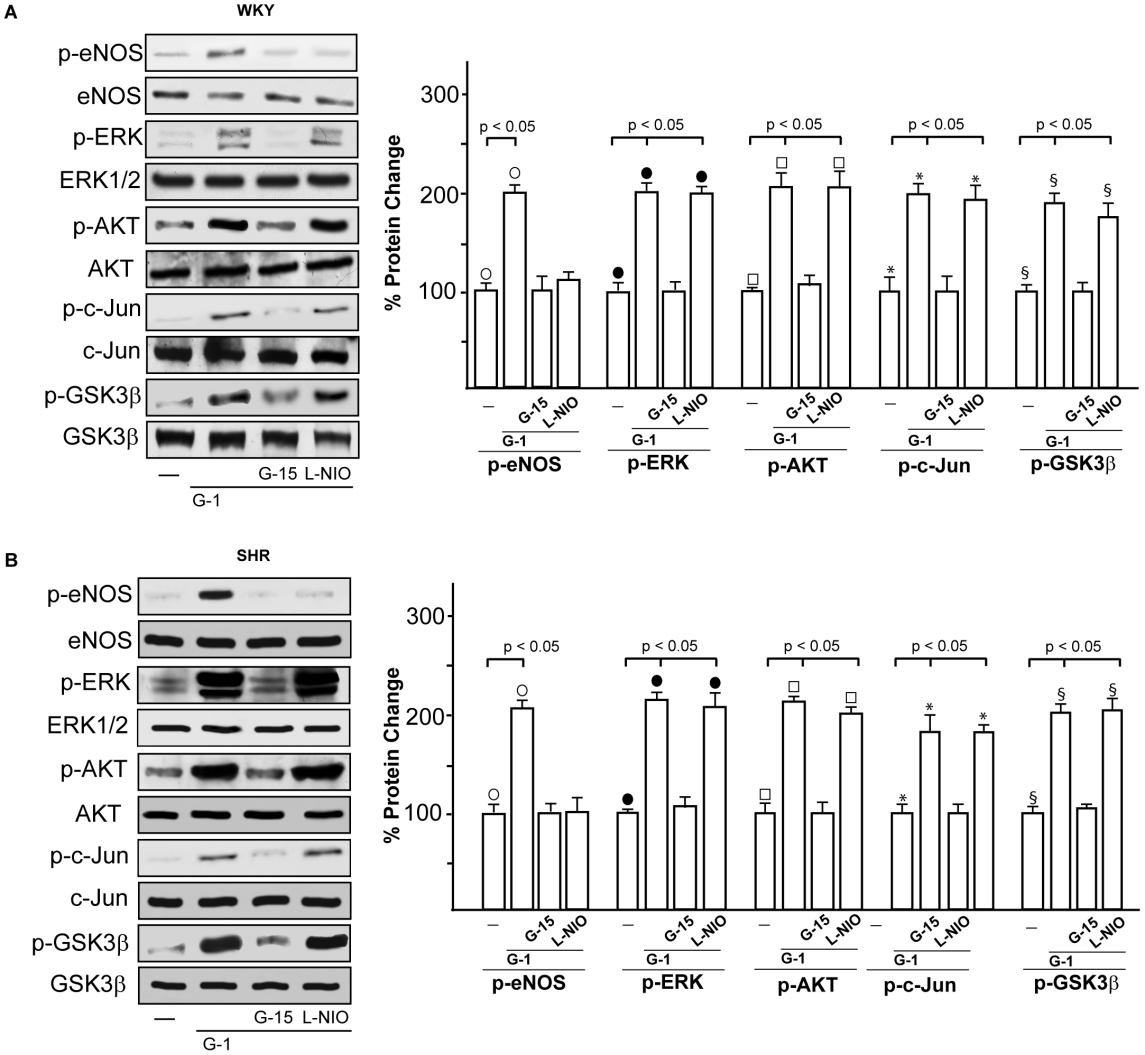
Activation of GPER-mediated signaling. (A) eNOS, ERK, AKT, c-Jun, and GSK3β phosphorylation in WKY left ventricular tissues perfused with vehicle (-), 1 nmol/L G-1 alone (2 h) and in combination with 100 nmol/L G15 (1 h) or 10 µmol/L L-NIO (1 h). The expression level of each phospho-protein was quantified by densitometry and normalized to the respective total protein content. Percentage changes were evaluated as the mean±SD of 5 experiments for each group. (○), (•), (□), (*), (§) p<0.05. (B) eNOS, ERK, AKT, c-Jun and GSK3β phosphorylation in SHR left ventricular tissues perfused with vehicle (-), 1 nmol/L G-1 alone (2 h) and in combination with 100 nmol/L G15 (1 h) or 10 µmol/L L-NIO (1 h). The expression level of each phospho-protein was quantified by densitometry and normalized to the respective total protein content. Percentage changes were evaluated as the mean±SD of 5 experiments for each group. (○), (•), (□), (*), (§) p<0.05.

On the basis of the aforementioned results, we aimed to evaluate whether the expression of ERα, ERβ and GPER could be altered by G-1 in WKY and SHR rat hearts preparations. To this end, WKY and SHR rat hearts were perfused with 1 nmol/L G-1 for 2 hours, however the expression of all receptors examined did not change at both mRNA (3A) and protein (3B) levels. Hence, the cardiotropic effects induced by G-1 did not involve alteration in ER and GPER expression. As GPER/eNOS signaling is involved in G-1 dependent cardiac modulation [25], we determined that in WKY rat (Fig. 3C) and SHR hearts (data not shown) the GPER selective antagonist G15 [34] and the selective eNOS inhibitor L-NIO [25] prevented the G-1 dependent negative inotropic and lusitropic effects (data not shown). On the basis of these findings, we aimed to characterize the transduction pathways activated by G-1 in order to evaluate the mechanisms involved in its cardiomodulatory action. In WKY rats (Fig. 4A) and SHR (Fig. 4B), the exposure to G-1 (1 nmol/L) induced eNOS phosphorylation, which was abrogated in presence of G15 and L-NIO (Fig. 4A–B). In addition, in WKY (Fig. 4A) and SHR (Fig. 4B) heart preparations exposed to G-1 (1 nmol/L), the activation of ERK1/2, AKT, c-Jun and GSK3β was abolished using G15 but not L-NIO.

### HIF-1α and CTGF expression in WKY and SHR hearts

**Figure 5:**
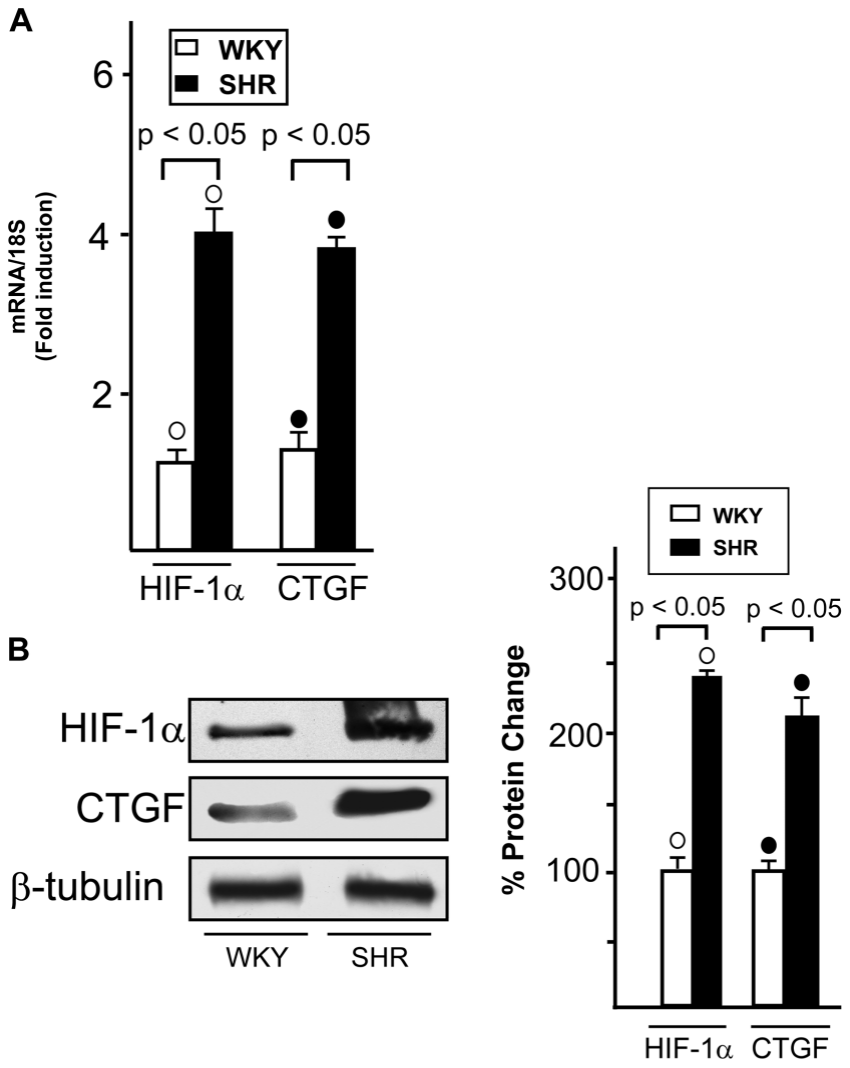
HIF-1α and CTGF expression in normotensive and hypertensive rat hearts. (A) Evaluation of HIF-1α and CTGF mRNA expression in WKY and SHR left ventricular tissue, as evaluated by Real Time PCR and normalization to 18S expression. Bars represent the mean±SD of 5 experiments for each group. (○), (•) p<0.05 for the expression in SHR vs WKY. (B) Evaluation of HIF-1α and CTGF protein expression in WKY and SHR ventricular tissue, protein expressions were normalized to β-tubulin. Percentage changes were evaluated as mean±SD of 5 experiments for each group. (○), (•) p<0.05 for the expression in SHR vs WKY.

**Figure 6:**
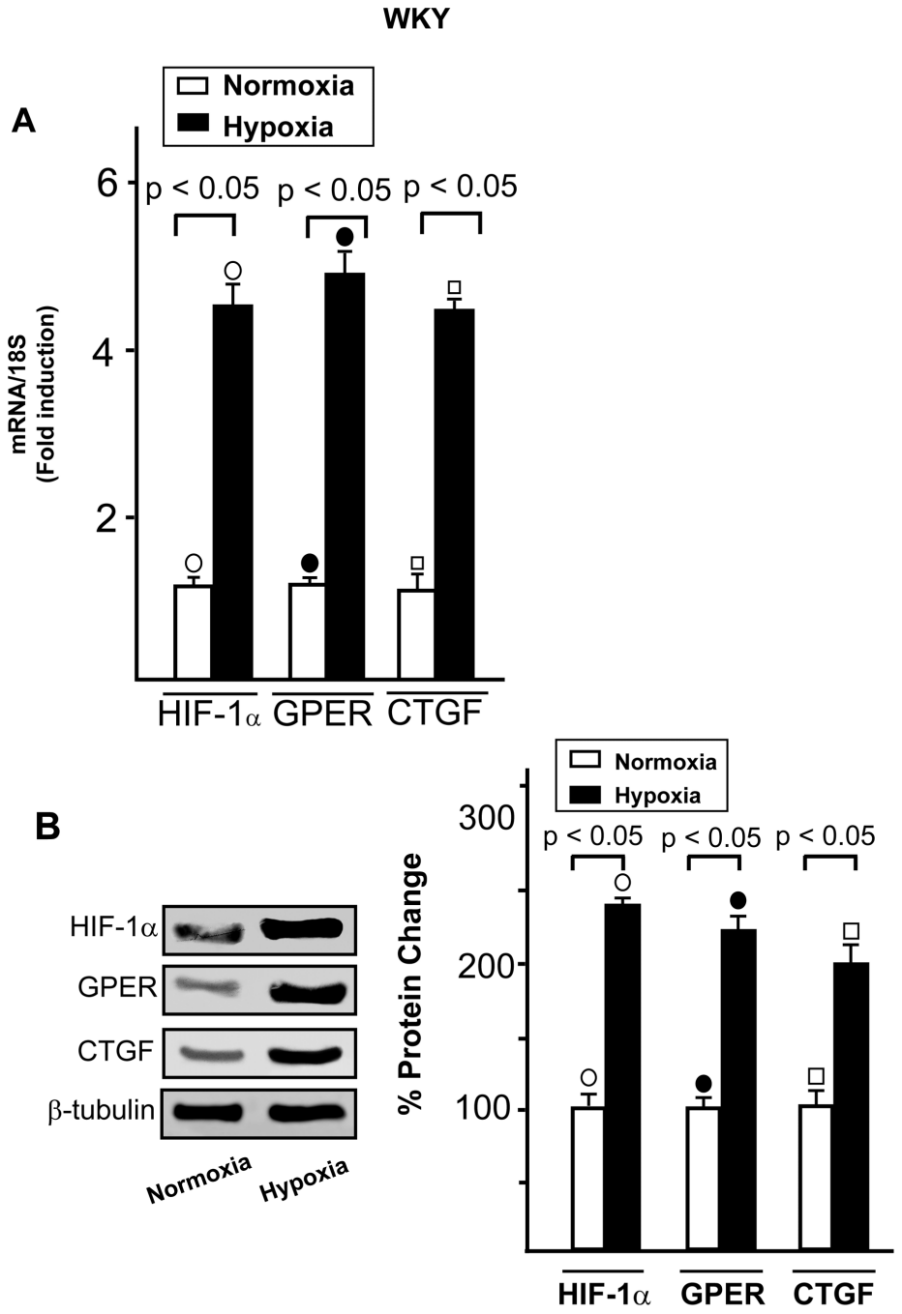
HIF-1α and CTGF expression in hypoxic cardiac preparations. (A) Evaluation of HIF-1α, GPER and CTGF mRNA expression by real time PCR in normoxic and hypoxic (1 h exposure to 40% pO_2_ levels) WKY left rat ventricle after normalization to 18S expression. Bars represent the mean±SD of 5 experiments for each group. (○), (•), (□) p<0.05 for the expression of hypoxic vs normoxic preparations. (B) Representative immunoblots showing HIF-1α, GPER and CTGF protein expression in normoxic and hypoxic (1 h exposure to 40% pO_2_ levels) male WKY rat left ventricle. Protein expressions were normalized to β-tubulin, percentage changes were evaluated as mean±SD of 5 experiments for each group. (○), (•), (□) p<0.05 for the expression of hypoxic vs normoxic preparations.

We have recently shown that GPER and CTGF are target genes of HIF-1α upon hypoxic conditions in cancer cells and cardiomyocytes [29]. Hence, we asked whether the increased levels of GPER in SHR compared to WKY rats (Fig. 1) could be paralleled by an enhancement of HIF-1α and CTGF expression. As shown in Figure 5, HIF-1α and CTGF were found up-regulated at both mRNA and protein levels in SHR compared to WKY heart preparations, suggesting that stressful hypertensive conditions activate the HIF-1α/GPER/CTGF signaling *in vivo* as we observed following the exposure to hypoxia in a different model system [29]. Accordingly, exposing to low oxygen tension (50% O_2_ for 1 h) WKY rat hearts, HIF-1α, GPER and CTGF mRNA and protein expressions increased in hypoxic preparations compared to normoxic homogenates (Fig. 6), while a similar experimental procedure performed in SHR hearts did not evidence a further up-regulation of these genes (data not shown) likely due to their elevated levels detectable before the exposure to hypoxia (Fig. 5). In WKY rats, exposure to low oxygen tension reduced LVP (LVP = 89+3 vs 45+4 mmHg, P<0.05), and coronary pressure (CP = 63±3 vs 43+7 mmHg, P<0.05), without significant changes in HR. A similar trend was observed in the SHR hypoxic heart.

**Figure 7:**
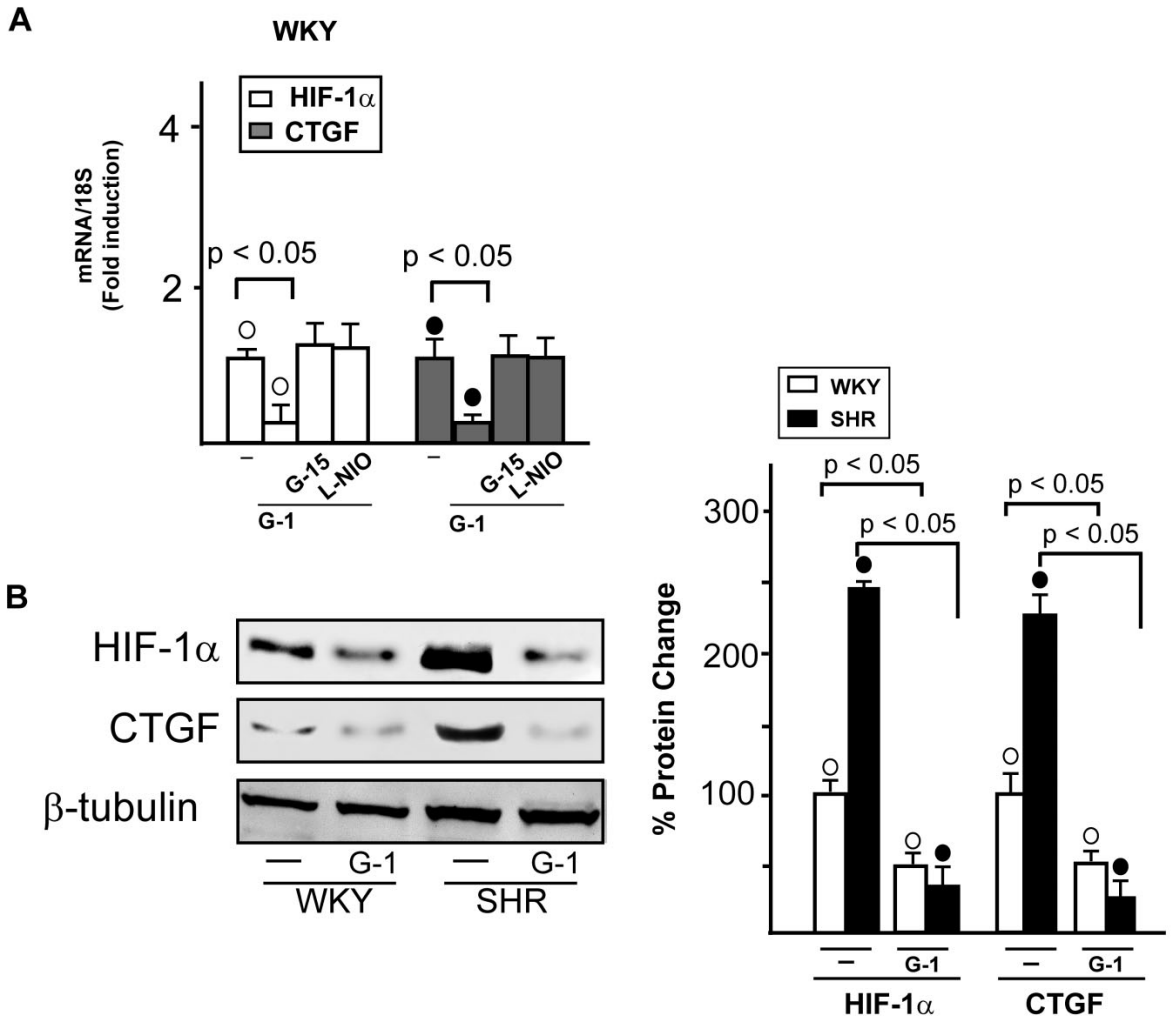
Involvment of GPER/eNOS signaling in the regulation of HIF-1α and CTGF expression. (A) Evaluation of HIF-1α and CTGF mRNA expression in WKY left ventricular tissues perfused with vehicle (-), 1 nmol/L G-1 alone (2 h) and in combination with 100 nmol/L G15 (1 h) or 10 µmol/L L-NIO (1 h), as evaluated by Real Time PCR and normalization to 18S expression. Bars represent the mean±SD of 5 experiments for each group. (○), (•) p<0.05. (B) Evaluation of HIF-1α and CTGF protein expression in WKY and SHR ventricular tissues treated with vehicle (-) or 1 nmol/L G-1 (2 h). Protein expressions were normalized to β-tubulin. Percentage changes were evaluated as mean±SD of 5 experiments for each group. (○), (•) p<0.05.

Then, we evaluated whether the GPER/eNOS transduction pathway may influence the expression of HIF-1α and CTGF, as the estrogenic signaling regulated HIF-1α and CTGF levels in a stressful environment [35]. In WKY (Fig. 7A) and SHR (data not shown) G-1 reduced the mRNA expression of both HIF-1α and CTGF, however this effect was abrogated in presence of G15 and L-NIO, suggesting that the GPER/eNOS transduction pathway is involved in the G-1 dependent decrease of HIF-1α and CTGF expression. Further supporting the abovementioned findings, G-1 reduced HIF-1α and CTGF protein levels in WKY and in SHR heart preparations (Fig. 7B). Altogether, these data suggest that the up-regulation of GPER may be included among the biological responses to stressful conditions like the regulation of HIF-1α and CTGF expression.

## Discussion

An increasing number of studies shows that GPER is involved in the signaling activated by estrogen and related compounds in numerous tissues [36]–[39], including the cardiovascular system [11], [24], [40]. As GPER is strongly expressed in the rat and human hearts [41], it may mediate the cardioprotective action elicited by estrogens together with or independently of ER [31], [42], [43]. Considering the growing interest addressed to better define the role exerted by GPER in cardiac physiopathology, we aimed to provide further insight into the mechanisms through which GPER may influence cardiac performance in SHR, which represent a useful model of essential hypertension. Our results demonstrate that both GPER mRNA and protein levels are increased in left ventricles from male SHR compared to male normotensive WKY rats. In isolated and Langendorff perfused rat hearts, the selective GPER ligand G-1 induced a reduction in contractility, as revealed by the decrement of LVP and +(LVdP/dt)max. This effect was more evident in SHR compared to WKY rats and independent of the chronotropism in both animal groups. In addition, the negative inotropism observed upon G-1 treatment in SHR and WKY rats was paralleled by negative lusitropic effects, as revealed by the reduction of -(LVdP/dt)max and the increase of T/-t. In spite of the remarkable effects on contractility and relaxation, G-1 did not affect coronary motility as only a slight, non-significant vasoconstriction was observed in WKY rats. On the basis of these observations, the elevated expression of GPER associated with a marked cardiodepression discovered in SHR, could elicit a protective role toward the stressful effects consequent to the high blood pressure. In agreement with this assumption, G-1 did not modify the endo-diastolic pressure, which is a well-known index of contracture. Hence, the aforementioned responses mediated by GPER could be beneficial in order to prevent and delay the hypertensive damages leading to cardiac hypertrophy and heart failure [44], [45]. In this regard, our findings highlight the valuable cardiac effects of GPER activation, according to a previous study showing that G-1 ameliorates diastolic dysfunction and reduces left ventricular hypertrophy in a model of salt-induced hypertensive cardiomyopathy [43]. Analogously, G-1 was reported to attenuate diastolic impairment and left ventricle remodeling in oophorectomized mRen2.Lewis rats, suggesting that GPER activation may mitigate the adverse effects of estrogen loss on left ventricle remodelling and diastolic damage [46].

Recalling previous reports which link estrogenic signals with eNOS activation [9], [25], in the present study the GPER/eNOS transduction pathway was shown to be involved in the negative inotropic and lusitropic effects induced by G-1 on the basis of the ability of the GPER antagonist G15 and the eNOS inhibitor L-NIO to abrogate these cardiotropic responses (see results section and figure 3). Moreover, we determined that the activation of diverse transduction cascades induced by G-1 lies upstream of the eNOS response, as G15 but not L-NIO prevented the phosphorylation of ERK1/2, AKT, GSK3β and c-Jun. Our current results are in line with diverse investigations showing that G-1 promotes cardiotropic actions through the activation of ERK/eNOS signaling and the PI3K/AKT transduction pathways [25], [26]. Further extending previous data linking c-Jun activity with NO production [47], we have also demonstrated that GPER is involved in the activation of c-Jun associated with eNOS phosphorylation. Next, we evidenced that G-1 perfusion induces also the activation of GSK3β, which is largely known to promote cell survival [48], [49]. Altogether, our findings suggest that G-1 triggers a cardioprotective signaling network involved in the “reperfusion injury salvage kinase (RISK) pathway” [50] which is activated by both ischaemic and pharmacological pre- and post-conditioning [50], [51], [52].

The hypoxic mediator HIF-1α and the fibrotic marker CTGF together with GPER were found expressed to a higher extent in SHR with compared to WKY left ventricles. Accordingly, an increased expression of these genes was detected in WKY exposed to low oxygen tension. It is well known that HIF-1, which is formed by the constitutive β subunit and the oxygen-sensitive α subunit [53], induces the transcription of factors involved in the adaptation to stressful condition [54]. Hence, under hypoxia [29] or hypertension as demonstrated in the present study, the up-regulation of GPER may be included among the biological adaptive responses to stressful microenvironment. In this regard, it should be mentioned that a functional cross-talk exists between hypertension and hypoxia, as hypoxic conditions which follow hypertension trigger HIF-1α expression and function [55]. In addition, hypoxia can provoke hypertension through diverse mechanisms mediated by HIF-1α [56] and leading to increased levels of catecholamines [57]. HIF-1 activation may also promote programmed cell death during hypoxia in a cell type specific manner [58], [59] contributing to the cardiac degeneration and progression toward heart failure [60]. Noteworthy, previous studies have shown that NO is able to inhibit HIF-1 activity and expression [61]–[64]. In line with these findings, our results indicate that the GPER/eNOS signaling mediates the down-regulation of both HIF-1α and its fibrotic target CTGF induced by G-1, particularly in the hypertensive rat heart model.

Our present findings let emerge an important role for GPER in the adaptation to stressful conditions, in line with previous data showing that G-1 ameliorates post-ischemic dysfunction and reduces infarct size after I/R [26]. Moreover, the ability of G-1 to preserve myocardial function when administered prior to global I/R has been associated with decreased myocardial inflammation [65], hence evidencing that the cardioprotective action mediated by GPER may involve multiple mechanisms which need to be further elucidated. In this context, it should be noted that we used male WKY and SHR that represent a unique experimental model as their exposure to estrogens is very low. This issue should be carefully considered as GPER-mediated function may be regulated by estrogens making difficult the interpretation of the biological responses. In addition, it should be mentioned that men and women in post-menopause present a higher risk to develop cardiovascular diseases respect to women before the menopause, that are exposed to elevated estrogen levels. Anyway, further studies are needed towards a better understanding of the potential of GPER to mediate beneficial effects in presence of a different hormone milieu.

Our previous study has demonstrated that the inhibition of ERα and ERβ abolishes the beneficial cardiac effects elicited by both E2 and G1 [25], suggesting a functional cross-talk between ERα, ERβ and GPER. ERα isoforms at 66 kD, 46 kD, and 36 kD have been identified, although the 66 kDa isoform has been widely characterized for its main role in mediating estrogenic stimuli [5], [66], [67]. G-1 has been shown to be able to activate the 36 kD isoform of ERα in human cancer cells [68], evidencing a possible contribution of this receptor isoform in triggering the biological responses to G-1. In this regard, it should be mentioned that similar evidence has not been reported in rat hearts, hence it should be argued that in our experimental model system the activation of GPER by G-1 is the mechanism involved in the cardiotropic effects observed, as further demonstrated by using the selective GPER antagonist G-15.

Collectively, the present study provides novel insight into the regulatory role played by GPER in stressful conditions characterized by an altered ventricular performance. Hence, GPER may be considered as a further therapeutic target in cardiac diseases on the basis of its involvement in myocardial inotropism and lusitropism as well as in the expression of the apoptotic and fibrotic factors HIF-1α and CTGF, respectively.

## Conclusion

In this study, we detected higher expression of GPER, the hypoxic mediator HIF-1α and the fibrotic mediator CTGF in left ventricles from SHR with respect to WKY rats. The GPER ligand G-1induced negative inotropic and lusitropic effects that could be considered as a protective action elicited by GPER. Moreover, the activation of the prosurvival/anti-apoptotic RISK pathway may further highlight the cardioprotection mediated by GPER upon pathophysiological conditions. Hence, the up-regulation of GPER expression may represent an adaptive response to stressful conditions such as hypertension and hypoxia. This may pave the way to analyze the therapeutic potential of the GPER-dependent transduction pathways in cardiac diseases.
